# Reversal of Dabigatran Using Idarucizumab in a Septic Patient with Impaired Kidney Function in Real-Life Practice

**DOI:** 10.1155/2016/1393057

**Published:** 2016-07-27

**Authors:** Thomas C. Sauter, Sina Blum, Michael Nagler, Fabian L. Schlittler, Meret E. Ricklin, Aristomenis K. Exadaktylos

**Affiliations:** ^1^Department of Emergency Medicine, Inselspital University Hospital, 3011 Bern, Switzerland; ^2^Department of Haematology and Central Haematology Laboratory, Inselspital University Hospital, 3011 Bern, Switzerland; ^3^Department of Cranio-Maxillofacial Surgery, Inselspital University Hospital, 3011 Bern, Switzerland

## Abstract

*Background*. Immediate reversal of anticoagulation is essential when facing severe bleeding or emergency surgery. Although idarucizumab is approved for the reversal of dabigatran in many countries, clinical experiences are lacking, particularly in special patient-populations such as sepsis and impaired renal function.* Case Presentation*. We present the case of a 67-year-old male septic patient with a multilocular facial abscess and chronic kidney disease (GFR 36.5 mL/min). Thrombin time (TT) and activated partial thromboplastin time (aPTT) 15 hours after the last intake of 150 mg dabigatran were both prolonged (>120 sec, resp., 61 sec), as well as unbound dabigatran concentration (119.05 ng/mL). Before immediate emergency surgery dabigatran was antagonised using idarucizumab 2 × 2.5 g. Dabigatran concentration was not detectable 10 min after idarucizumab administration (<30 ng/mL). TT and aPTT time were normalised (16.2 sec, resp., 30.2 sec). Sepsis was controlled after surgery and kidney function remained stable. In the absence of postoperative bleeding, dabigatran was restarted 36 hours after admission.* Conclusion*. Idarucizumab successfully reversed the effect of dabigatran in real-life practice in a patient with sepsis and renal impairment and allowed emergency surgery with normal haemostasis. Efficacy and safety in real-life practice will nevertheless require prospective registries monitoring.

## 1. Introduction

Immediate reversal of anticoagulation is essential when facing severe bleeding or emergency surgery. Even though the use of direct oral anticoagulants is rapidly increasing, no antidote for the reversal of dabigatran was available for use in clinical practice so far [1, 2]. Haemodialysis of dabigatran is possible but time consuming and not available in all cases [3, 4]. In 2015, the FDA and EMA approved idarucizumab (Praxbind^©^), a monoclonal antibody, for the specific inactivation of dabigatran by direct binding. However, approval was based on a single-arm prospective observational study with laboratory measures as primary endpoint only [5]. Idarucizumab is rapidly eliminated by the kidney with a mean plasma concentration decreased by 80% from the peak level 4 hours after administration [5]. Elimination in patients with severely impaired renal function is unclear. Efficacy of idarucizumab in clinical practice with regard to clinical endpoints is unknown, in particular in special populations such as renal impairment or sepsis.

## 2. Case Presentation

The 67-year-old male patient was admitted to our emergency department because of swelling in his right cheek since the previous day. He had a history of atrial fibrillation, which had been treated with dabigatran, 150 mg twice daily (last dose 15-16 hours before presentation), and suffered from chronic renal impairment due to type II diabetes mellitus and hypertension. The febrile patient was haemodynamically stable on admission. The physical examination revealed significant hot and painful right facial swelling. The laboratory results on admission were as follows: white blood count 17.7 Giga/L; C-reactive protein 234 mg/L; creatinine 165 *μ*mol/L (GFR 36.5 mL/min); and unbound dabigatran concentration as measured with diluted thrombin time (dTT; Hyphen Hemoclot, Neuville-sur-Oise, France) 119.05 ng/mL. The activated partial thromboplastin time (aPTT) and thrombin time were prolonged (61 sec, resp., <120 sec). For a summary of coagulation tests, see Table 1. We administered 2.2 g i.v. Co-Amoxicillin and computed tomography was performed to plan abscess surgery.

As the patient was becoming increasingly septic and haemodynamically unstable despite volume substitution, emergency surgery was indicated.

We administered a 2.5 g infusion of idarucizumab, followed by a second infusion of 2.5 g idarucizumab after 10 minutes. No side effects were reported by the patient.

Immediately after the second dose, coagulation testing was repeated and aPTT as well as thrombin time were found to be normal; dabigatran concentration was below detection limit (<30 ng/mL; see Table 1). Thirty minutes after idarucizumab application, enoral incision of the multilocular abscess was performed, with irrigation and insertion of Penrose and Easy Flow drainage. There was no relevant blood loss and no other complications. The surgical team rated the intraoperative bleeding as being “as expected.”

The patient could be managed postoperatively on an ordinary surgical ward with rapid clinical improvement. Coagulation tests were repeated 24 hours after administration of idarucizumab (Table 1). Postoperative kidney function improved after treatment of infection (creatinine 115 *μ*mol/L, GFR 56 mL/min, day 6). As there was no postoperative bleeding, dabigatran was restarted 36 hours after surgery and administration of idarucizumab. The patient was discharged from hospital without any complications.

## 3. Discussion

We report the successful administration of idarucizumab to a septic patient with chronic kidney disease, in order to facilitate emergency surgery for facial abscess.

Although our patient presented about 15-16 hours after the last 150 mg dose of dabigatran, coagulation tests were still markedly prolonged and a relevant dabigatran concentration was present. Given the patient's rapid clinical deterioration and his impaired renal function, we decided to administer the antidote, rather than waiting for the dabigatran to be cleared. As only 13 patients in the RE-VERSE AD study (33%) had creatinine clearance <50 mL/min, experience with application of idarucizumab and restarting of dabigatran in patients with impaired renal function is limited [5].

The RE-VERSE AD trail included two patients with abscesses only, in the suprapubic and scrotal locations [5]. However, bleeding in abscess surgery can be critical, particularly in oral surgery where haemorrhages may compromise the airway and lead to aspiration that requires invasive airway management. In our patient, reversal of anticoagulation led to immediate normalisation of all coagulation tests. Normal intraoperative haemostasis, as reported by the surgical team in our case, is consistent with the 92% of patients in the RE-VERSE AD trail [5].

Twenty-four hours after surgery and administration of idarucizumab, we found that aPTT and thrombin time were mildly prolonged again. Most probably, these changes are caused by redistribution of unbound dabigatran not detected by the diluted thrombin time. Even in patients with severe renal impairment idarucizumab is most likely to be cleared at 24 h hours and therefore cannot bind redistributed dabigatran anymore. It has been previously reported that even very low doses of 25 ng/mL dabigatran (below the detection limit of our test, <30 ng/mL) may lead to immeasurably high thrombin times [6]. This prolongation in the coagulation tests did not lead to any clinical bleeding in our patient but may cause confusion. It is important to point out that the aPTT is likely to be prolonged in the presence of dabigatran, but the degree of prolongation does not correlate well with the level of anticoagulant activity.

In-hospital acute kidney injury is common, especially in patients with sepsis (2–18% of all hospital inpatients) and needs close monitoring of renal function to determine whether and at which dosage therapy with dabigatran can be continued [7, 8]. In our patient, the kidney function improved after treatment of infection and he could be discharged with dabigatran into the care of his general practitioner for close monitoring.

## 4. Conclusion

Idarucizumab reversed the effect of dabigatran treatment successfully in real-life practice in a patient with sepsis and renal impairment and allowed emergency surgery with normal haemostasis. As there is limited experience with idarucizumab efficacy and safety in real-life practice shall be monitored in prospective registries.

## Figures and Tables

**Table 1:** Coagulation tests.

Parameter	Standard values	Before idarucizumab	10 minutes after 2 × 2.5 g idarucizumab	24 hours after idarucizumab
INR		1.56	1.23	1.10
aPTT (sec)	25.0–36.0	61	30.2	41.5
Thrombin time (sec)	11.5–19.4	>120	16.2	52.9
Fibrinogen (g/L)	1.75–3.75	7.11	7.11	7.09
Dabigatran (ng/mL)		119.05	<30.0	<30.0

Activated partial thromboplastin time (aPTT); international normalised ratio (INR).
